# Development and evaluation of competency-based curriculum for continuing professional development among military nurses: a mixed methods study

**DOI:** 10.1186/s12909-022-03846-1

**Published:** 2022-11-16

**Authors:** Huijuan Ma, Aifang Niu, Li Sun, Yu Luo

**Affiliations:** 1 School of Nursing, Third Military Medical University/Army Medical University, No. 30 Gaotanyan Street, Shapingba District, Chongqing, P.R. China; 2 Health Management Center, First Affiliated Hospital, Army Medical University, No. 30 Gaotanyan Street, Shapingba District, Chongqing, P.R. China

**Keywords:** Competency-based curriculum, Continuing professional development, Military nurse

## Abstract

**Background:**

Continuing professional development (CPD) is essential for career progression and maintaining military nursing competency. A well-designed CPD programme can improve the effectiveness of transforming knowledge and skills in healthcare organisations. This study aimed to develop a competency-based CPD curriculum for military nurses in China and evaluate its effectiveness from a developmental pilot study.

**Methods:**

In phase one, a two-round Delphi was conducted to design a competency-based curriculum of CPD based on a clinical ladder model among military nurses. In phase two, the curriculum of one CPD programme was redesigned, and a pilot quasi-experiment was conducted to evaluate the effectiveness of this programme.

**Results:**

A competency-based curriculum was developed for primary, intermediate, and senior titles, respectively. The trainees’ overall satisfaction with the redesigned CPD programme was 100%. The four themes in the qualitative data were: 1) learning motivation and learning barriers; 2) professional growth; 3) role model promoted career planning; 4) learning environment mattered.

**Conclusion:**

This study developed a competency-based curriculum for continuing professional development among military nurses that can be used in designing CPD programmes. Competency-based curriculum can be utilised in the CPD activities to facilitate the improvement of nursing competency.

**Supplementary Information:**

The online version contains supplementary material available at 10.1186/s12909-022-03846-1.

## Background

Nurses constitute the largest part of the healthcare workforce and are fundamental to ensuring the delivery of high-quality care within healthcare organisations [1]. Nurses are expected to maintain nursing competency to fulfil their roles [2], and competency is reportedly related to effective job performance, quality of care, and patient safety [3, 4]. Career advancement provides opportunities for nurses to enhance their competencies by participating in professional development programmes [5]. Continuing professional development (CPD), as part of career advancement, is essential for career progression and maintenance of nursing competency [6, 7].

CPD among nurses is valued by many countries and the International Council of Nurses [8–10]. Nurses in China are encouraged to participate in CPD activities and obtain at least 25 CPD credit points (approximately 75 hours) each year, whereas nurses in the United Kingdom (UK) have to undertake 12 hours of CPD annually [11, 12]. The advantages of CPD are twofold: for healthcare organisations, CPD can promote health outcomes by improving the quality of care; for nurses, CPD can promote their motivation and satisfaction towards work [13, 14]. In this context, nursing administrators and scholars have focused on the design and implementation of effective CPD.

CPD among nurses is organised in various modes, and competency-based CPD as well as the clinical ladder model are frequently used [14, 15]. Competency-based CPD is based on a model for healthcare quality improvement that views competency as a dynamic process in which health professionals need CPD activities to sustain their competency by acquiring new knowledge and skills and make progress towards becoming experts in one’s profession [14, 16]. Competency-based CPD conceptualises competency-based education (CBE) and CPD, which focuses on measurable competency improvement to meet the needs of healthcare and patient outcomes [16, 17]. The clinical ladder model was introduced by Zimmer in 1972 and has been used extensively in CPD along with the theory ‘Novice to Expert’ [18, 19]. The clinical ladder model divides nursing competencies into multiple stages and clarifies the expected competencies at each stage [20]. The clinical ladder model has many advantages in the nursing profession, including enhancement of professional development, improvement of retention, and sustainment of competency [20, 21].

Military nurses are responsible for providing healthcare in hospitals during peacetime and in relief operations during natural disasters or epidemics [22]; thus, their competencies are more complex. The onion model, which is an enriched competency model with different interrelated layers, is frequently used to provide a comprehensive perspective for exploring competencies and designing multilevel training [23, 24]. Based on the onion model, a competency model for military nurses in general hospitals was developed by our research team, covering four main categories: motive, traits, self-identity of dual roles, as well as knowledge, skills, and abilities [24]. Owing to the multiple responsibilities of military nurses, they are required to sustain competencies by participating in the CPD programme. The above study of developing competency model for military nurses provides theoretical guidance for competency-based CPD by gaining a better understanding of military nurses’ competencies. As such, the practice scope of military nurses presents a unique challenge for the implementation of the CPD programme.

CPD among military nurses is also valued by military organisations in many countries [25]. In the United States (US), the Army Nursing Leader Capabilities Map, along with the corresponding training programmes, were designed to sustain the competencies of Army Nursing leaders from three levels according to the complexity of management skill application: tactical/direct, operational, and strategic [26, 27]. In the UK, the Defence Operational Nursing Competency (DONC) was developed to provide guidelines for the professional development of military nurses [28]. In China, a stratified training programme explored in a military hospital was effective in improving the comprehensive quality of military nurses [29]. CPD programmes in military organisations were organised according to the clinical ladder model. However, there have been no reports in the literature on the implementation of competency-based CPD among military nurses. CBE has been frequently applied in health professional education in the past decade [30]. A competency-based curriculum for CPD would be a suitable strategy to sustain the competency of military nurses in complex work environments, and the clinical ladder model could be used to guide curriculum development. Therefore, a developmental pilot design and mixed methods were conducted to explore the curriculum of CPD among military nurses based on the competency model of military nurses developed by our research team.

## Methods

### Research design

The study protocol was approved by the Medical Ethics Committee of the Army Medical University in Chongqing, China. A two-phase procedure was implemented in this study. In Phase 1, a two-round Delphi was conducted to design a competency-based curriculum based on a clinical ladder model for military nurses. In Phase 2, the curriculum of one CPD programme undertaken by the organisation of the search team was redesigned based on the findings of Phase 1, and a pilot quasi-experiment was conducted to evaluate the effectiveness of this redesigned curriculum.

### Curriculum development

Based on the competency model of military nurses in general hospitals developed by our research team [24], Pubmed, Embase, CNKI, and Wanfang were searched to collect literature including corresponding training courses of each competency in the competency model. This literature review was conducted to build an item pool of competency-based curricula for the continuing professional education of military nurses. After several group discussions, a competency-based curriculum was developed (see Additional File 1), which was composed of nine modules and 46 training contents. The nine modules were clinical nursing, military nursing, critical thinking, nursing interpersonal communication and etiquette, nursing teaching, nursing research, nursing management, political literacy, and military quality.

Experts were invited to rate the level of importance of the 46 training contents of the above nine modules for three different professional titles of nurses (primary title, intermediate title, and senior title) on a 5-point Likert scale from 1 (least important) to 5 (most important). They were also invited to express their opinions on the training content. A panel of experts was selected from among military nursing education, military nursing management, clinical nursing, and military medical service education professionals. The following inclusion criteria were used in this process: (1) bachelor’s degree or higher; (2) professional experience exceeding 10 years and engaged in the field of military nursing and military medicine with a solid theoretical foundation; and (3) consent to participate. Purposive sampling was conducted to ensure that the national panel had diverse expertise. The researchers prepared a list of 23 potential participants. The target experts were contacted to determine their willingness to participate in this study, and 22 experts who met the eligibility criteria consented to participate.

Each expert who consented to participate in this study completed a questionnaire. They were asked to give feedback within two weeks, with a reminder for those who failed to provide feedback within the given time. After receiving all the completed questionnaires from the expert panel, the data collected were analysed, including the mean, standard deviation, coefficient of variation, Kendall coefficient, and suggestions of experts, following which the questionnaire for the next round was developed. When consensus was reached among the panel, the data collection process was stopped, and the final competency-based curriculum of CPD was developed. The trustworthiness of the Delphi study was enhanced by credibility, transferability, confirmability, and dependability through procedures including high response rate and diversity of participated experts [31, 32].

Data analysis was performed using IBM SPSS Statistics for Windows version 22.0. The following variables were included in this study: demographics, approval rate (percentage of statements rated as 4 or 5 on a 1–5 significance Likert scale), and the coefficient of variation (CV). A statement was excluded if it met one of the following criteria: the mean value of the significance score was less than 3.5, approval rate was less than 70%, and CV was more than 0.25.

### Implementation

The research team chose one CPD programme to conduct an empirical study. This programme was open for military nurses of junior and intermediate professional titles, and those who wanted to participate could sign up. Owing to the shortfall of training resources, the research team could not divide trainees of different professional titles into two groups. Therefore, the curriculum of this CPD programme was redesigned by merging the competency-based curricula for primary and intermediate titles. A pilot quasi-experiment was conducted to evaluate the effectiveness of this curriculum because it was not ethical to conduct a randomised controlled study in the same programme [33]. Data were collected in two phases: pre- and post-training.

The pre-test questionnaire included two parts: a demographic questionnaire (e.g. gender, marital status, professional title, age, education status, position, year of clinical career) and the professional competency scale for military nurses (PCSMN) [34]. The PCSMN is a self-assessment tool and comprises 65 items in four domains: clinical nursing (15 items), military nursing (17 items), professional ability (20 items), and comprehensive quality (13 items). Each item was rated on a 5-point Likert scale, with higher scores indicating higher levels of competency. The Cronbach’s alpha of PCSMN was 0.983.

The post-test collected both quantitative and qualitative data. Quantitative data were collected using the PCSMN and satisfaction questionnaire, which included the satisfaction of trainees with the training content, teachers, and environment. Qualitative data were collected through individual semi-structured interviews to explore the experience of the trainees participating in this training programme. The interview outline included learning motivation, learning gains, and leaning environment. Each interview lasted for approximately one hour, and notes were taken in each interview.

The above mixed methods design was used to collect both quantitative and qualitative date to explore the effectiveness of the CPD programme. Quantitative data of PCSMN was used to detect the quantitative change of competency, and qualitative data was used to detect trainees’ changes that were not well captured by quantitative techniques. As learning motivation and environmental factors can influence adult learners based on adult learning theories [35], qualitative data was also used to explore trainees’ experience and perception of the programme.

A quantitative data analysis was performed using IBM SPSS Statistics for Windows 22.0. Descriptive statistics were used to analyse the demographic characteristics of the participants, mean PCSMN score, and satisfaction rate of trainees. The PCSMN scores followed a normal distribution based on the Shapiro-Wilk test, and paired t-tests were used to analyse the pre- and post-training differences in PCSMN. Qualitative data analysis was performed using qualitative content analysis, which focused on both manifest and latent content [36]. Once each interview was completed, we read the transcribed text several times and examined the whole interview as the unit of analysis to outline the meaning of the units and phrases. We assigned codes to each meaning unit and subsequently classified the codes into categories and subcategories by comparing codes based on differences and similarities [36]. Saturation was reached when no new codes emerged, and two additional interviews were conducted to ensure saturation [37]. To enhance the trustworthiness of the qualitative study, we enhanced credibility, dependability, confirmability, and transferability through procedures including prolonged engagement, external audits, and the provision of quotes with rich descriptions [38, 39].

## Results

### Curriculum development

#### The first round

Initially, 23 experts consented to participate, of which 22 (95%) completed the questionnaire in the first round. Their demographic characteristics are presented in Table 1. The expert panel was composed of military nursing managers, military nursing educators, military nurses, and military medical service educators, with the means of age and working years being 42.1 ± 5.03 and 22.4 ± 7.31, respectively.

**Table 1:** Demographic characteristics of experts

	Participants	Number	Percent
Qualification	Bachelor	10	45.5
	Master	3	13.6
	Doctor	9	40.9
Professional title	Middle	1	4.5
	High	15	68.2
	Senior	6	22.3
Working years	≤15	5	22.7
	16–25	11	50.0
	≥26	6	27.3
Profession	Nursing management	5	22.7
	Nursing education	7	31.8
	Clinical nursing	8	36.4
	Military medical service	2	9.1
Age	≤40	8	36.4
	41–50	13	59.1
	≥51	1	4.5

For the primary title, 27 contents including surgical cooperation, clinical nursing teaching design, evidence-based nursing, and nursing research management were deleted, as they met the criteria for deletion; for the intermediate title, 13 contents including fundamental nursing, interpersonal communication, nursing research project application, and nursing research management were deleted, as they met the criteria as well; for the senior title, 17 contents including fundamental nursing, critical thinking, humanistic care, and ethics in nursing research were deleted for the same reason (see Additional File 1).

As suggested by the experts, ‘Surgical cooperation’ was deleted because of a lack of generality. The experts also advised that the curriculum be refined according to the different professional titles of the military nurses. Therefore, the questionnaire for the second-round survey was developed and refined according to the results of the first-round survey, which included three competency-based curricula for primary, intermediate, and senior titles. The curriculum of the primary title included three modules (military nursing, clinical nursing, and comprehensive quality) and 34 teaching contents. The intermediate curriculum title included five modules (military nursing, clinical nursing, nursing teaching and research, nursing management, and comprehensive quality) and 38 teaching contents. The senior curriculum title included four modules (military nursing, nursing teaching and research, nursing management, and comprehensive quality) and 40 teaching contents (see Additional File 2).

#### The second round

In the second round, 22 experts completed the questionnaire. Regarding the primary title, three contents were deleted as they met the deletion criteria. As suggested by experts, two contents of the intermediate title were deleted, as they were covered in the curriculum of the primary title. Furthermore, two contents of the senior title were deleted, as they overlapped with the curricula of the primary and intermediate titles (see Additional File 2). Based on the results of the second round, a competency-based curriculum of the CPD programme for primary, intermediate, and senior titles was developed.

### Implementation

#### Curriculum of continuing professional education programme

The CPD programme curriculum covered the training content of five modules: military nursing, clinical nursing, nursing teaching and research, nursing management, and comprehensive quality. To arrange the courses more clearly, the curriculum was redesigned into three modules: military nursing, clinical nursing, nursing teaching, and research (see Additional File 3). For nursing management, two training contents were designed and arrayed in the military and clinical nursing modules, respectively. As for comprehensive quality, several contents, including a team building activity and face-to-face communication with the winners of the International Nightingale Medal, were designed and arrayed in three different modules. This programme were conducted during November 1–30, 2021, with 23 nurses enrolled. The teaching team comprised the International Nightingale Medal winners, international wound treatment experts, doctoral supervisors, and senior professors in military nursing.

#### Quantitative results

The survey sample consisted of 22 nurses aged 35.5 ± 5.2 years (range 24–45), who had 13.0 ± 5.58 years of work experience (range 1–28). The other demographic characteristics are presented in Table 2.

**Table 2:** Demographic characteristics of participants in quantitative study

Variable	*n*	%
Gender		
Female	22	100.0
Male	0	0.00
Age		
Less than 30 years old	4	18.2
31 to 40 years old	15	68.2
More than 40 years old	3	13.6
Year of working as nurse		
Less than 10 years	5	22.7
11 to 20 years	15	68.2
More than 20 years	2	9.1
Current Level of Education		
College degree or lower	1	4.5
Bachelors	21	95.5
Professional Title		
Senior nurse	10	45.5
Nurse in charge	12	54.5

After analysing the satisfaction survey, it was found that trainees’ overall satisfaction with the training programme was 100%. Trainees’ satisfaction with the training contents, teachers, organisation, and environment was also 100% (see Table 3).

**Table 3:** Satisfaction of trainees

	very dissatisfied	fairly dissatisfied	neither satisfied nor dissatisfied	fairly satisfied	very satisfied
Content of military nursing	0(0%)	0(0%)	1(4.5%)	11(50.0%)	10(45.5%)
Content of nursing teaching and research	0(0%)	0(0%)	1(4.5%)	5(22.7%)	16(72.7%)
Content of clinical nursing	0(0%)	0(0%)	1(4.5%)	6(27.3%)	15(68.2%)
Training content	0(0%)	0(0%)	0(0%)	4(18.2%)	18(81.8%)
Training teacher	0(0%)	0(0%)	0(0%)	1(4.5%)	21(95.5%)
Training organization	0(0%)	0(0%)	0(0%)	2(9.1%)	20(90.9%)
Training equipment	0(0%)	0(0%)	1(4.5%)	7(31.8%)	14(63.6%)
Training environment	0(0%)	0(0%)	0(0%)	3(13.6%)	19(86.4%)
Overall satisfaction	0(0%)	0(0%)	0(0%)	3(13.6%)	19(86.4%)

**Fig. 1:**
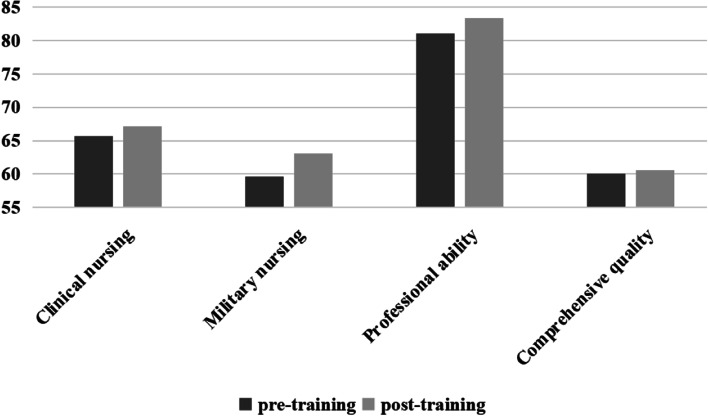
Comparison of PCSMN score before and after training

After analysing the PCSMN survey, it was found that although the difference between the pre- and post-training scores was not statistically significant (*P* > 0.05), the overall score of PCSMN and each dimension was higher in the post-training than in the pre-training period (Table 4 and Fig. 1).

**Table 4:** Comparison of PCSMN score before and after training

Dimension	Pre-training	Post-training	*t*	*p*
Clinical nursing	65.7 ± 5.13	67.2 ± 5.33	−1.53	0.141
Military nursing	59.6 ± 12.58	63.1 ± 10.73	−1.11	0.280
Professional ability	81.1 ± 8.72	83.4 ± 8.73	−1.01	0.284
Comprehensive quality	60.1 ± 4.79	60.6 ± 4.95	−0.58	0.566
Overall score of competency	266.6 ± 24.46	274.3 ± 24.39	−1.30	0.207

### Qualitative results

The interview sample consisted of 14 nurses aged 35.8 ± 4.46 years (range 27–42) who had 13.4 ± 4.07 years of work experience (range 5–22). The characteristics of the other samples are listed in Table 5.

**Table 5:** Demographic characteristics of participants in qualitative study

Variable	*n*	%
Gender		
Female	14	100.0
Male	0	0.0
Age		
Less than 30 years old	3	21.4
31 to 40 years old	9	64.3
More than 40 years old	2	14.3
Year of working as nurse		
Less than 10 years	3	21.4
11 to 20 years	10	71.4
More than 20 years	1	7.2
Current Level of Education		
College degree or lower	1	7.1
Bachelors	13	92.9
Professional Title		
Senior nurse	6	42.9
Nurse in charge	8	57.1

We identified the following four themes in the qualitative data: learning motivation and learning barriers, professional growth, role model promoted career planning, and staff, organisation, and learning climate impacted trainees in positive way.

#### Learning motivation and learning barriers

Learning motivation includes personal interest, clinical practice needs, self-improvement needs, and expectations from the organisation. The trainees mentioned that they were interested in training content that could be used in clinical practice. The trainees also stated that they had a sense of recognition when appointed by the organisation to participate in the training programme.

*‘I wanted to go out to study as much as possible, and then get in touch with new knowledge, and then I came here for this purpose’. (P01).*

Learning barriers include family and work factors. The trainees mentioned that they did not want to leave for training because they were worried about their children. They also spoke about the heavy workloads that hindered their participation.

*‘Actually, I want to come, however, because of heavy workload and lack of manpower, many nurses cannot participate in this training programme’. (P09).*

#### Professional growth

Professional growth of trainees included they gained knowledge, skills, and abilities during this programme and wanted to apply these into practice. The trainees mentioned that they were exposed to new knowledge, and improved their communication abilities.

*‘ We updated our knowledge, and I gained a lot’. (P04).*

The trainees mentioned that they wanted to apply what they have learned in the area of psychological nursing, humanistic care, palliative care, and wound care in clinical practice and daily life.

*‘The intensive care and pressure ulcer care I learned this time can be used clinically’. (P02).*

#### Role model promoted career planning

This training programme invited two winners of the International Nightingale Medal to communicate face-to-face with the trainees. Trainees mentioned that the winners of the International Nightingale Medal are the leaders of the career path, not only firm in their belief in the nursing profession but also help they plan career paths.

*‘I think the head nurse makes us feel more determined, and I learned how to lead my team and nurses well. In fact, it is quite rewarding’. (P06).*

#### Learning environment mattered

Learning environment including staff, organisation, and learning climate positively impacted trainees. Trainees mentioned that the teachers were warm and enthusiastic about teaching and that the teaching organisation unit had carefully planned the courses. The trainees stated that they were full of energy during training.

*‘Generally speaking, I think the teachers must have worked hard in the design of the curriculum. Although this is a short-term training, the teaching staff is experienced. I am moved by the careful arrangement of the training plan’. (P09).*

## Discussion

In this study, a competency-based curriculum for CPD among military nurses of primary, intermediate, and senior titles was developed through a modified Delphi study based on the competency model of military nurses in general hospitals developed by our research team [24]. Meanwhile, the CPD programme among military nurses with primary and intermediate titles was redesigned, and the effectiveness of its implementation was evaluated from the trainees’ perspective.

The importance of CPD for nurses is widely recognised, and research on nurses’ experiences regarding CPD is well documented in the literature [13, 40]. Past research has shown that nurses valued CPD and considered it fundamental for professionalism and lifelong learning [41]. Nurses at varying career stages had different perceptions of CPD, and it is necessary to scientifically design and implement CPD from the perspective of career advancement [13]. Owing to the complexity of military nurses’ competencies and their multiple responsibilities, well-designed CPD programmes among military nurses are necessary. This study was innovative in developing the CPD curriculum from the perspective of CBE and career advancement, which could be a suitable approach to lifelong learning [41].

This study also evaluated the effectiveness of a competency-based curriculum for CPD among military nurses. The results of the quantitative study showed that trainees were satisfied with the training content, organisation, and environment of the CPD programme. The competency level of military nurses could be improved through the four-week CPD programme. Previous research has shown that there is no standardised pre-deployment training curriculum for military nurses [42], and the curriculum in this study could also provide evidence for future research in this area.

Research on nurses’ experiences of CPD are well documented in the literature. However, reports of military nurses’ experiences with CPD are scant. This study detailed the learning motivation, barriers, and experiences of military nurses with regard to CPD. In this study, the motivation of trainees was consistent with past research on nurses, including the desire for career progression, willingness to learn, and desire to provide high-quality care [40]. Meanwhile, barriers to participation in CPD among trainees were mainly childcare and heavy workload, which were also consistent with past research [43]. Moreover, trainees mentioned that they gained knowledge, skills, and abilities, and wanted to apply what they had learned into practice. Therefore, the effectiveness of competency-based CPD and its future potential for nurses is clear.

This study has several limitations. First, the methodology of the Delphi study has its own shortcomings, such as the difficulty in generalising the results to a wider population [44]. To improve generalisability, an intensive literature review was conducted when developing the competency model and competency-based curriculum [24, 34]. Second, a competency-based curriculum was implemented as a pilot study among nurses of primary and intermediate titles. In the future, observational research can be implemented to further test its effectiveness by comparing long-term outcomes. Third, maturation and experimenter effects, as major problems of internal validity in the simple quasi-experiment design [45], can not be discounted. A stronger quasi-experimental design can be carried out to compare with previous cohort studies that undertook different training methods and to compare this model with other alternative.

## Conclusions

CPD is essential for career progression and maintaining competency in nurses, and a well-designed CPD programme can improve the effectiveness of transforming knowledge and skills in healthcare organisations. This study developed a competency-based curriculum for CPD among military nurses, which can be used in designing CPD programmes in the worldwide. Competency-based curriculum can be utilised in the CPD activities to facilitate the improvement of nursing competency. Further more rigorous research would be needed to evaluate the effectiveness of competency-based CPD activities on the basis of this current competency-based curriculum.

## Supplementary Information

**Additional file 1.**

**Additional file 2.**

**Additional file 3.**

## Data Availability

The datasets used and/or analysed during the current study are available from the corresponding author on reasonable request.

## Abbreviations

CBE: competency-based education
CPD: continuing professional development
CV: coefficient of variation
DONC: Defence Operational Nursing Competency
PCSMN: Professional Competency Scale for Military Nurses
