# Supplementary material for: Development and evaluation of competency-based curriculum for continuing professional development among military nurses: a mixed methods study
Source: BMC Med Educ. 2022 Nov 16;22:793. doi: 10.1186/s12909-022-03846-1 (PMC9667581; doi:10.1186/s12909-022-03846-1)
Supplement: Supplementary file 1 — Additional file 1. [file 12909_2022_3846_MOESM1_ESM.docx]

**Additional File 1 Results of the first round**

| Dimension | Module | Content | Primary title | | | | Intermediate title | | | | Senior title | | | | Note |
| --- | --- | --- | --- | --- | --- | --- | --- | --- | --- | --- | --- | --- | --- | --- | --- |
|  |  |  | Mean | SD | CV | Approval rate | Mean | SD | CV | Approval rate | Mean | SD | CV | Approval rate |  |
| Clinical nursing knowledge and skills | 1.1 Clinical nursing | 1.1.1 Fundamental nursing | 4.95 | 0.21 | 0.04 | 100% | 4.05 | 1.17 | 0.29 | 77% | 3.55 | 1.30 | 0.37 | 50% | #& |
|  |  | 1.1.2 Specialist nursing | 4.45 | 0.74 | 0.17 | 86% | 4.45 | 0.51 | 0.11 | 100% | 4.14 | 0.99 | 0.24 | 68% | & |
|  |  | 1.1.3 Emergency critical care | 4.50 | 0.67 | 0.15 | 91% | 4.73 | 0.46 | 0.10 | 100% | 4.41 | 0.91 | 0.21 | 73% | & |
|  |  | 1.1.4 Nursing safety and legal knowledge | 4.86 | 0.35 | 0.07 | 100% | 4.27 | 0.98 | 0.23 | 73% | 4.18 | 0.91 | 0.22 | 68% | #& |
| Military nursing knowledge and skills | 1.2 Military nursing | 1.2.1 Combat casualty care | 4.73 | 0.55 | 0.12 | 95% | 4.14 | 1.04 | 0.25 | 77% | 4.00 | 0.93 | 0.23 | 68% | & |
|  |  | 1.2.2 Organisation and implementation of trauma care | 3.55 | 0.86 | 0.24 | 41% | 4.59 | 0.50 | 0.11 | 100% | 4.86 | 0.35 | 0.07 | 100% | * |
|  |  | 1.2.3 Common trauma care | 4.59 | 0.67 | 0.15 | 91% | 4.73 | 0.46 | 0.10 | 100% | 4.55 | 0.67 | 0.15 | 91% |  |
|  |  | 1.2.4 Trauma care in a special environment | 3.86 | 0.89 | 0.23 | 64% | 4.59 | 0.59 | 0.13 | 95% | 4.68 | 0.57 | 0.12 | 95% | * |
|  |  | 1.2.5 Trauma care owing to special weapons | 3.73 | 0.83 | 0.22 | 59% | 4.59 | 0.59 | 0.13 | 95% | 4.68 | 0.57 | 0.12 | 95% | * |
|  |  | 1.2.6 Nutritional support | 3.95 | 0.95 | 0.24 | 77% | 4.50 | 0.74 | 0.16 | 95% | 4.45 | 0.67 | 0.15 | 91% | * |
|  |  | 1.2.7 Surgical cooperation | 3.82 | 1.01 | 0.26 | 64% | 4.27 | 0.88 | 0.21 | 82% | 3.95 | 0.72 | 0.18 | 73% | *&# |
|  |  | 1.2.8 Psychological care | 4.14 | 0.94 | 0.23 | 73% | 4.64 | 0.58 | 0.13 | 95% | 4.82 | 0.50 | 0.10 | 95% | * |
|  |  | 1.2.9 Health and epidemic prevention technology | 4.55 | 0.67 | 0.15 | 91% | 4.27 | 0.98 | 0.23 | 73% | 4.23 | 1.07 | 0.25 | 73% | #& |
| Professional ability | 2.1 Critical thinking | 2.1 Critical thinking | 4.41 | 0.67 | 0.15 | 91% | 4.32 | 0.89 | 0.21 | 73% | 4.41 | 0.91 | 0.21 | 73% | #& |
|  | 2.2 Nursing interpersonal communication and etiquette | 2.2.1 Interpersonal communication | 4.77 | 0.43 | 0.09 | 100% | 4.09 | 0.92 | 0.23 | 73% | 3.86 | 1.04 | 0.27 | 50% | #& |
|  |  | 2.2.2 Nursing etiquette | 4.55 | 0.96 | 0.21 | 91% | 3.86 | 1.08 | 0.28 | 68% | 3.91 | 1.11 | 0.28 | 59% | #& |
|  |  | 2.2.3 Multicultural nursing | 4.18 | 1.10 | 0.26 | 73% | 4.27 | 0.94 | 0.22 | 91% | 4.14 | 1.08 | 0.26 | 73% | *& |
|  |  | 2.2.4 Humanistic care | 4.68 | 0.57 | 0.12 | 95% | 4.64 | 0.66 | 0.14 | 91% | 4.09 | 1.11 | 0.27 | 68% | & |
|  | 2.3 Nursing teaching | 2.3.1 Clinical nursing teaching design and method | 3.14 | 0.71 | 0.23 | 23% | 4.41 | 0.91 | 0.21 | 95% | 4.86 | 0.35 | 0.07 | 100% | * |
|  |  | 2.3.2 Clinical nursing teaching ward round | 3.36 | 1.00 | 0.30 | 41% | 4.50 | 0.60 | 0.13 | 95% | 4.77 | 0.43 | 0.09 | 100% | * |
|  |  | 2.3.3 Teaching of clinical nursing skills | 3.50 | 0.80 | 0.23 | 50% | 4.64 | 0.58 | 0.13 | 95% | 4.55 | 0.74 | 0.16 | 95% | * |
|  |  | 2.3.4 Clinical nursing health education | 4.36 | 0.95 | 0.22 | 77% | 4.55 | 0.67 | 0.15 | 91% | 4.05 | 0.90 | 0.22 | 73% | & |
|  |  | 2.3.5 Evaluation of nursing clinical teaching | 3.14 | 0.83 | 0.27 | 27% | 4.36 | 0.79 | 0.18 | 91% | 4.91 | 0.29 | 0.06 | 100% | * |
|  | 2.4 Nursing research | 2.4.1 Literature search skill | 3.82 | 1.18 | 0.31 | 64% | 4.68 | 0.57 | 0.12 | 95% | 4.32 | 0.89 | 0.21 | 73% | *& |
|  |  | 2.4.2 Nursing research design | 3.50 | 1.01 | 0.29 | 55% | 4.55 | 0.74 | 0.16 | 95% | 4.36 | 0.90 | 0.21 | 73% | *& |
|  |  | 2.4.3 Nursing research project application | 3.27 | 0.94 | 0.29 | 45% | 3.95 | 0.84 | 0.21 | 73% | 4.86 | 0.47 | 0.10 | 95% | *# |
|  |  | 2.4.4 Writing and publishing of nursing papers | 3.59 | 1.14 | 0.32 | 64% | 4.55 | 0.60 | 0.13 | 95% | 4.68 | 0.57 | 0.12 | 95% | * |
|  |  | 2.4.5 Evidence-based nursing and evidence transformation | 3.59 | 1.18 | 0.33 | 59% | 3.91 | 1.02 | 0.26 | 68% | 4.82 | 0.50 | 0.10 | 95% | *# |
|  |  | 2.4.6 Nursing technology application | 3.27 | 1.03 | 0.32 | 41% | 3.95 | 0.95 | 0.24 | 73% | 4.91 | 0.29 | 0.06 | 100% | *# |
|  |  | 2.4.7 Ethics and norms in nursing research | 3.95 | 1.13 | 0.29 | 64% | 4.64 | 0.58 | 0.13 | 95% | 4.41 | 0.91 | 0.21 | 73% | *& |
|  | 2.5 Nursing management | 2.5.1 Management theory and principle | 2.91 | 1.02 | 0.35 | 27% | 4.23 | 0.75 | 0.18 | 91% | 4.82 | 0.50 | 0.10 | 95% | * |
|  |  | 2.5.2 Nursing resource management | 3.18 | 1.10 | 0.34 | 32% | 4.41 | 0.67 | 0.15 | 91% | 4.77 | 0.53 | 0.11 | 95% | * |
|  |  | 2.5.3 Nursing quality management | 3.27 | 1.08 | 0.33 | 41% | 4.55 | 0.60 | 0.13 | 95% | 4.91 | 0.29 | 0.06 | 100% | * |
|  |  | 2.5.4 Nursing clinical education management | 3.09 | 0.92 | 0.30 | 27% | 4.41 | 0.59 | 0.13 | 95% | 4.86 | 0.47 | 0.10 | 95% | * |
|  |  | 2.5.5 Nursing information management | 3.41 | 1.05 | 0.31 | 45% | 3.95 | 0.72 | 0.18 | 73% | 4.82 | 0.50 | 0.10 | 95% | *# |
|  |  | 2.5.6 Nursing research management | 3.00 | 1.07 | 0.36 | 32% | 3.95 | 0.65 | 0.17 | 77% | 4.91 | 0.29 | 0.06 | 100% | *# |
|  |  | 2.5.7 Leadership | 2.55 | 0.80 | 0.31 | 5% | 3.77 | 0.53 | 0.14 | 73% | 4.95 | 0.21 | 0.04 | 100% | *# |
|  |  | 2.5.8 Organisation and implementation of medical support | 2.91 | 0.87 | 0.30 | 9% | 3.86 | 0.71 | 0.18 | 77% | 4.86 | 0.47 | 0.10 | 95% | *# |
| Comprehensive quality | 3.1 Political literacy | 3.1.1 Military professional ethics | 4.64 | 0.90 | 0.19 | 95% | 4.50 | 0.96 | 0.21 | 91% | 4.59 | 0.73 | 0.16 | 86% |  |
|  |  | 3.1.2 Military history | 4.45 | 0.96 | 0.22 | 91% | 4.41 | 0.85 | 0.19 | 86% | 4.50 | 0.74 | 0.16 | 86% |  |
|  |  | 3.1.3 Current affairs and political education | 4.41 | 1.01 | 0.23 | 86% | 4.41 | 0.85 | 0.19 | 86% | 4.50 | 0.74 | 0.16 | 86% |  |
|  |  | 3.1.4 Traditional education | 4.45 | 0.96 | 0.22 | 91% | 4.41 | 0.85 | 0.19 | 86% | 4.45 | 0.74 | 0.17 | 86% |  |
|  |  | 3.1.5 Core value | 4.68 | 0.89 | 0.19 | 95% | 4.59 | 0.80 | 0.17 | 91% | 4.64 | 0.66 | 0.14 | 91% |  |
|  |  | 3.1.6 Military regulation | 4.68 | 0.89 | 0.19 | 95% | 4.32 | 1.04 | 0.24 | 86% | 4.23 | 0.87 | 0.21 | 73% | & |
|  | 3.2 Military quality | 3.2.1 Military basis | 4.50 | 0.91 | 0.20 | 95% | 4.41 | 0.96 | 0.22 | 91% | 4.41 | 0.85 | 0.19 | 86% |  |
|  |  | 3.2.2 Physical training | 4.50 | 0.80 | 0.18 | 91% | 4.41 | 0.85 | 0.19 | 86% | 4.32 | 1.04 | 0.24 | 82% |  |
| Note: * represent content of primary title was deleted as they met the criteria for deletion or suggested by the expert panel; #represent content of intermediate title was deleted as they met the criteria for deletion or suggested by the expert panel; & represent content of senior title was deleted as they met the criteria for deletion or suggested by the expert panel. | | | | | | | | | | | | | | | |
