# Supplementary material for: Development and evaluation of competency-based curriculum for continuing professional development among military nurses: a mixed methods study
Source: BMC Med Educ. 2022 Nov 16;22:793. doi: 10.1186/s12909-022-03846-1 (PMC9667581; doi:10.1186/s12909-022-03846-1)
Supplement: Supplementary file 2 — Additional file 2. [file 12909_2022_3846_MOESM2_ESM.docx]

**Additional File 2 Results of the second round**

| **Layer** | **Module** | **Content** | **Mean** | **SD** | **CV** | **Approval rate** | **Note** |
| --- | --- | --- | --- | --- | --- | --- | --- |
| Primary title | Military nursing | 1.1 Organisation and implementation of field medical centre | 4.64 | 0.73 | 0.16 | 91% |  |
|  |  | 1.2 Characteristics and basic requirements of field nursing work | 4.64 | 0.49 | 0.11 | 100% |  |
|  |  | 1.3 Casualty identification and assessment | 4.91 | 0.29 | 0.06 | 100% |  |
|  |  | 1.4 Haemostasis, bandaging, fixation, handling, ventilation | 5.00 | 0.00 | 0.00 | 100% |  |
|  |  | 1.5 Basic life support | 5.00 | 0.00 | 0.00 | 100% |  |
|  |  | 1.6 Field blood transfusion and field infusion | 4.95 | 0.21 | 0.04 | 100% |  |
|  |  | 1.7 Rules for the treatment of war wounds | 4.86 | 0.35 | 0.07 | 100% |  |
|  |  | 1.8 Assessment and management of shock patient | 4.91 | 0.29 | 0.06 | 100% |  |
|  |  | 1.9 Treatment and nursing of war injuries | 4.77 | 0.53 | 0.11 | 95% |  |
|  |  | 1.10 Treatment and nursing of various injured parts | 4.77 | 0.43 | 0.09 | 100% |  |
|  |  | 1.11 Mass casualty treatment | 4.64 | 0.58 | 0.13 | 95% |  |
|  |  | 1.12 Injury treatment and nursing in field internal medicine | 4.55 | 0.51 | 0.11 | 100% |  |
|  |  | 1.13 Hygiene and epidemic prevention personal protection technology | 4.91 | 0.29 | 0.06 | 100% |  |
|  |  | 1.14 Management of acute infectious disease | 4.50 | 0.74 | 0.16 | 86% |  |
|  |  | 1.15 Sanitary protection against damage from special weapons | 4.45 | 0.74 | 0.17 | 86% |  |
|  | Clinical nursing | 1.16 Advances in the fields of intravenous infusion, pipeline care, airway care, nutritional support, and wound stoma care | 4.55 | 0.80 | 0.18 | 91% |  |
|  |  | 1.17 Advances in psychological nursing | 3.95 | 0.84 | 0.21 | 73% | * |
|  |  | 1.18 Advances in palliative care practice | 3.82 | 0.85 | 0.22 | 64% | * |
|  |  | 1.19 Advances in acute and critical care nursing | 4.00 | 0.93 | 0.23 | 68% | * |
|  |  | 1.20 Nursing laws and regulations and medical dispute | 4.68 | 0.57 | 0.12 | 95% |  |
|  |  | 1.21 Nursing risk prevention and emergency response | 4.82 | 0.50 | 0.10 | 95% |  |
|  |  | 1.22 Sudden changes in common diseases and emergency treatment | 4.77 | 0.53 | 0.11 | 95% |  |
|  |  | 1.23 Emergency primary crisis identification and emergency plan drill | 4.82 | 0.39 | 0.08 | 100% |  |
|  |  | 1.24 Teamwork in critically ill patient | 4.82 | 0.39 | 0.08 | 100% |  |
|  |  | 1.25 Nursing interpersonal communication skill | 4.73 | 0.55 | 0.12 | 95% |  |
|  |  | 1.26 Nursing professional image and etiquette | 4.59 | 0.59 | 0.13 | 95% |  |
|  |  | 1.27 Nursing humanities | 4.68 | 0.48 | 0.10 | 100% |  |
|  |  | 1.28 Humanistic care technology and practice | 4.59 | 0.50 | 0.11 | 100% |  |
|  |  | 1.29 Health education | 4.82 | 0.39 | 0.08 | 100% |  |
|  | Comprehensive quality | 1.30 Political education | 4.82 | 0.39 | 0.08 | 100% |  |
|  |  | 1.31 Basic knowledge of medical support | 4.68 | 0.48 | 0.10 | 100% |  |
|  |  | 1.32 Military topography | 4.00 | 0.98 | 0.24 | 77% |  |
|  |  | 1.33 Joint operation | 3.82 | 0.96 | 0.25 | 68% |  |
|  |  | 1.34 Physical training | 4.64 | 0.58 | 0.13 | 95% |  |
| Intermediate title | Military nursing | 2.1 Classification and characteristics of war wounded | 4.82 | 0.39 | 0.08 | 100% |  |
|  |  | 2.2 Organisation and evacuation of the wounded | 4.86 | 0.35 | 0.07 | 100% |  |
|  |  | 2.3 Common trauma care | 4.95 | 0.21 | 0.04 | 100% |  |
|  |  | 2.4 Treatment and nursing of various injured parts | 4.86 | 0.35 | 0.07 | 100% |  |
|  |  | 2.5 Mass casualty treatment | 4.86 | 0.35 | 0.07 | 100% |  |
|  |  | 2.6 Injury treatment and nursing in field internal medicine | 4.86 | 0.35 | 0.07 | 100% |  |
|  |  | 2.7 Nursing care of combat casualties in special environment | 4.77 | 0.43 | 0.09 | 100% |  |
|  |  | 2.8 Nursing care of weapon injuries | 4.77 | 0.43 | 0.09 | 100% |  |
|  |  | 2.9 Nutritional assessment and parenteral nutrition support | 4.82 | 0.39 | 0.08 | 100% |  |
|  |  | 2.10 Nutritional support for critically ill/wounded | 4.86 | 0.35 | 0.07 | 100% |  |
|  |  | 2.11 Combat stress response nursing | 4.68 | 0.48 | 0.10 | 100% |  |
|  | Clinical nursing | 2.12 Advances in the fields of intravenous infusion, pipeline care, airway care, nutritional support, and wound stoma care | 4.82 | 0.39 | 0.08 | 100% |  |
|  |  | 2.13 Advances in psychological nursing | 4.73 | 0.46 | 0.10 | 100% |  |
|  |  | 2.14 Advances in palliative care practice | 4.68 | 0.48 | 0.10 | 100% |  |
|  |  | 2.15 Advances in acute and critical care nursing | 4.91 | 0.29 | 0.06 | 100% |  |
|  |  | 2.16 Intercultural Nursing Theory | 4.36 | 0.66 | 0.15 | 91% |  |
|  |  | 2.17 Humanistic care | 4.77 | 0.43 | 0.09 | 100% |  |
|  | Nursing teaching and research | 2.18 Teaching methods of clinical nursing | 4.91 | 0.29 | 0.06 | 100% |  |
|  |  | 2.19 Clinical nursing teaching ward round | 4.91 | 0.29 | 0.06 | 100% |  |
|  |  | 2.20 Teaching methods and skills of clinical nursing skills | 4.91 | 0.29 | 0.06 | 100% |  |
|  |  | 2.21 Forms and skills of health education | 4.82 | 0.39 | 0.08 | 100% |  |
|  |  | 2.22 Evaluation of nursing intern | 4.64 | 0.49 | 0.11 | 100% |  |
|  |  | 2.23 Literature search method | 4.91 | 0.29 | 0.06 | 100% |  |
|  |  | 2.24 Nursing research design | 4.77 | 0.43 | 0.09 | 100% |  |
|  |  | 2.25 Data collection and data analysis | 4.91 | 0.29 | 0.06 | 100% |  |
|  |  | 2.26 Writing and publishing of nursing papers | 4.91 | 0.29 | 0.06 | 100% |  |
|  |  | 2.27 Ethics and norms in nursing research | 4.91 | 0.29 | 0.06 | 100% |  |
|  | Nursing management | 2.28 Basic principles and theories of management | 4.82 | 0.39 | 0.08 | 100% |  |
|  |  | 2.29 Nursing human resource management | 4.68 | 0.48 | 0.10 | 100% |  |
|  |  | 2.30 Management of nursing equipment | 4.82 | 0.39 | 0.08 | 100% |  |
|  |  | 2.31 Nursing fund management | 4.45 | 0.60 | 0.13 | 95% |  |
|  |  | 2.32 Time management | 4.68 | 0.57 | 0.12 | 95% |  |
|  |  | 2.33 Nursing quality management | 4.82 | 0.39 | 0.08 | 100% |  |
|  | Comprehensive quality | 2.34 Political education | 4.86 | 0.35 | 0.07 | 100% |  |
|  |  | 2.35 Basic knowledge of medical support | 4.68 | 0.48 | 0.10 | 100% | * |
|  |  | 2.36 Military topography | 4.36 | 0.58 | 0.13 | 95% | * |
|  |  | 2.37 Joint operation | 4.41 | 0.59 | 0.13 | 95% |  |
|  |  | 2.38 Physical training | 4.68 | 0.48 | 0.10 | 100% |  |
| Senior title | Military nursing | 3.1 Health and combat readiness of military hospitals | 4.86 | 0.35 | 0.07 | 100% |  |
|  |  | 3.2 Wartime medical service organisation and command | 4.73 | 0.55 | 0.12 | 95% |  |
|  |  | 3.3 Organising the coordination of medical support | 4.91 | 0.29 | 0.06 | 100% |  |
|  |  | 3.4 Organisational safeguard action management | 4.91 | 0.29 | 0.06 | 100% |  |
|  |  | 3.5 Classification and characteristics of war wounded | 4.86 | 0.47 | 0.10 | 95% |  |
|  |  | 3.6 Organisation and evacuation of war wounded | 4.91 | 0.29 | 0.06 | 100% |  |
|  |  | 3.7 Treatment and nursing of war injury | 4.86 | 0.35 | 0.07 | 100% |  |
|  |  | 3.8 Treatment and nursing of various injured parts | 4.86 | 0.35 | 0.07 | 100% |  |
|  |  | 3.9 Mass casualty treatment | 4.86 | 0.35 | 0.07 | 100% |  |
|  |  | 3.10 Injury treatment and nursing in field internal medicine | 4.77 | 0.43 | 0.09 | 100% |  |
|  |  | 3.11 Nursing care of combat casualties in special environment | 4.82 | 0.39 | 0.08 | 100% |  |
|  |  | 3.12 Nursing care of weapon injuries | 4.82 | 0.39 | 0.08 | 100% |  |
|  |  | 3.13 Nutritional assessment and parenteral nutrition support | 4.86 | 0.35 | 0.07 | 100% |  |
|  |  | 3.14 Nutritional support for critically ill/wounded | 4.91 | 0.29 | 0.06 | 100% |  |
|  |  | 3.15 Combat stress response nursing | 4.77 | 0.43 | 0.09 | 100% |  |
|  | Nursing teaching and research | 3.16 Teaching design of clinical nursing course | 4.82 | 0.39 | 0.08 | 100% |  |
|  |  | 3.17 Development and implementation of clinical teaching plan | 4.91 | 0.29 | 0.06 | 100% |  |
|  |  | 3.18 Organisation and management of teaching activities | 4.91 | 0.29 | 0.06 | 100% |  |
|  |  | 3.19 Safety management in clinical nursing teaching | 4.77 | 0.43 | 0.09 | 100% |  |
|  |  | 3.20 Organisation and management of nursing teaching ward round | 4.86 | 0.35 | 0.07 | 100% |  |
|  |  | 3.21 Evaluation and feedback of nursing clinical teaching | 4.82 | 0.50 | 0.10 | 95% |  |
|  |  | 3.22 Application for nursing research projects | 4.95 | 0.21 | 0.04 | 100% |  |
|  |  | 3.23 Writing and publishing of nursing papers | 4.82 | 0.39 | 0.08 | 100% |  |
|  |  | 3.24 Evidence-based nursing and evidence transformation | 4.95 | 0.21 | 0.04 | 100% |  |
|  |  | 3.25 Nursing technology application | 4.91 | 0.29 | 0.06 | 100% |  |
|  | Nursing management | 3.26 Hospital management structure and strategic management | 4.77 | 0.53 | 0.11 | 95% |  |
|  |  | 3.27 Reform and development trend of nursing service model | 4.77 | 0.43 | 0.09 | 100% |  |
|  |  | 3.28 Management innovation and leadership | 4.77 | 0.43 | 0.09 | 100% |  |
|  |  | 3.29 Nursing economics and cost control | 4.68 | 0.48 | 0.10 | 100% |  |
|  |  | 3.30 Management communication and team building | 4.95 | 0.21 | 0.04 | 100% |  |
|  |  | 3.31 Human resource management and performance management | 4.91 | 0.29 | 0.06 | 100% |  |
|  |  | 3.32 Stress management | 4.86 | 0.35 | 0.07 | 100% |  |
|  |  | 3.33 Nursing quality improvement | 4.91 | 0.29 | 0.06 | 100% |  |
|  |  | 3.34 Nursing information management | 4.86 | 0.35 | 0.07 | 100% |  |
|  |  | 3.35 Nursing research project management | 4.86 | 0.35 | 0.07 | 100% |  |
|  | Comprehensive quality | 3.36 Political education | 4.82 | 0.39 | 0.08 | 100% |  |
|  |  | 3.37 Basic knowledge of medical support | 4.55 | 0.67 | 0.15 | 91% | * |
|  |  | 3.38 Military topography | 4.32 | 0.57 | 0.13 | 95% | * |
|  |  | 3.39 Joint operation | 4.32 | 0.65 | 0.15 | 91% | * |
|  |  | 3.40 Physical training | 4.68 | 0.48 | 0.10 | 100% |  |
| Note: * represent content was deleted as they met the criteria for deletion or suggested by the expert panel. | | | | | | | |
