# Supplementary material for: Development and evaluation of competency-based curriculum for continuing professional development among military nurses: a mixed methods study
Source: BMC Med Educ. 2022 Nov 16;22:793. doi: 10.1186/s12909-022-03846-1 (PMC9667581; doi:10.1186/s12909-022-03846-1)
Supplement: Supplementary file 3 — Additional file 3. [file 12909_2022_3846_MOESM3_ESM.docx]

**Additional File 3 Curriculum of continuing professional education programme**

| Module | Content |
| --- | --- |
| Military nursing | Team building activity |
|  | Organisation and implementation of field medical centre |
|  | Characteristics and basic requirements of field nursing work |
|  | Nursing management of new infectious disease hospital |
|  | Construction and application of emergency medical rescue mode for major public health emergencies |
|  | Psychological stress and intervention in wartime |
|  | Control and resuscitation of extension injury in special environment |
|  | Military training injury protection |
|  | Tactical Conbat Casualty Care |
|  | Prolonged field care |
|  | Comprehensive skills competition of field nursing in special environmental medical support |
| Nursing teaching and research | Nurse career planning |
|  | Clinical nursing curriculum design |
|  | Nursing research project design |
|  | Nursing paper writing and publishing |
|  | Evidence-based nursing and literature search |
|  | Action structure workshop |
| Clinical nursing | Clinical nursing rounds training |
|  | Progress in critically ill clinical nursing |
|  | The role of nurses in dementia risk control and intervention |
|  | Advances in wound care |
|  | Establishment and application of chronic kidney disease case management and long-term care model |
|  | New model of clinical nursing management |
|  | Evidence-based and practice of nutritional care after gastric cancer surgery |
|  | Psychological distress management |
|  | Palliative care practice |
|  | Application of positive psychology in clinical nursing |
|  | Sandplay therapy training |
|  | Mindfulness training |
|  | Face-to-face communication with the winners of the International Nightingale Medal |
